# A Co-Culture Model of the Human Respiratory Tract to Discriminate the Toxicological Profile of Cationic Nanoparticles According to Their Surface Charge Density

**DOI:** 10.3390/toxics9090210

**Published:** 2021-08-31

**Authors:** Yasmin Arezki, Juliette Cornacchia, Mickaël Rapp, Luc Lebeau, Françoise Pons, Carole Ronzani

**Affiliations:** Laboratoire de Conception et Application de Molécules Bioactives, Faculté de Pharmacie, UMR 7199, CNRS-Université de Strasbourg, 67400 Illkirch, France; y.arezki@unistra.fr (Y.A.); jcornacchia@unistra.fr (J.C.); rappm@unistra.fr (M.R.); llebeau@unistra.fr (L.L.); pons@unistra.fr (F.P.)

**Keywords:** co-culture model, multi-endpoint approach, nanoparticles, carbon dots, nanotoxicology, lung toxicity, inflammatory potential

## Abstract

This study aimed at discriminating with sensitivity the toxicological effects of carbon dots (CDs) with various zeta potential (ζ) and charge density (Q_ek_) in different cellular models of the human respiratory tract. One anionic and three cationic CDs were synthetized as follows: CD-COOH (ζ = −43.3 mV); CD-PEI600 (Q_ek_ = 4.70 µmol/mg; ζ = +31.8 mV); CD-PEHA (Q_ek_ = 3.30 µmol/mg; ζ = +29.2 mV) and CD-DMEDA (Q_ek_ = 0.01 µmol/mg; ζ = +11.1 mV). Epithelial cells (A549) and macrophages (THP-1) were seeded alone or as co-cultures with different A549:THP-1 ratios. The obtained models were characterized, and multiple biological responses evoked by CDs were assessed in the mono-cultures and the best co-culture model. With 14% macrophages, the 2:1 ratio co-culture best mimicked the in vivo conditions and responded to lipopolysaccharides. The anionic CD did not induce any effect in the mono-cultures nor in the co-culture. Among the cationic CDs, the one with the highest charge density (CD-PEI600) induced the most pronounced responses whatever the culture model. The cationic CDs of low charge density (CD-PEHA and CD-DMEDA) evoked similar responses in the mono-cultures, whereas in the co-culture, the three cationic CDs ranked according to their charge density (CD-PEI600 > CD-PEHA > CD-DMEDA), when taking into account their inflammatory effect. Thus, the co-culture system developed in this study appears to be a sensitive model for finely discriminating the toxicological profile of cationic nanoparticles differing by the density of their surface charges.

## 1. Introduction

The production of manufactured nanoparticles (NPs) raises genuine concerns about health risks, especially by inhalation [1,2]. In vivo studies have provided valuable information on NP deposition and toxicological effects in the lung [3,4]. Inhaled NPs were shown to deposit all throughout the airways, and to particularly accumulate in the alveoli [4], where they are internalized by epithelial cells and macrophages [5]. Furthermore, lung tissue inflammation and remodeling were reported in rodents upon inhalation or lung instillation of NPs [5,6,7]. However, the huge diversity of NPs in terms of physicochemical characteristics, including chemical composition, size, shape, and surface charge means that not all NPs can be evaluated by in vivo approaches [8]. Indeed, animal studies are costly, time-consuming and should be reduced in accordance with the 3R principle. Moreover, cellular and molecular mechanisms involved in NP toxicity cannot be easily studied in vivo [9]. Therefore, over the last years, several in vitro models have been developed to investigate on the safety of NPs [10,11].

Both mono- and co-culture models are currently used to assess the toxicological effects of NPs in the lung. Mono-culture systems are mainly based on bronchial (Calu-3, NCI-H292 or 16-HBE) or alveolar (A549) epithelial cell lines and/or on immune cells, particularly macrophages (especially activated THP-1), as they are the main cell targets for inhaled NPs [12]. These mono-culture systems are commonly used to screen a large number of NPs [13] and to determine their toxicity mechanisms [14]. They are also used to propose toxicological endpoints to be considered for NP safety assessment, e.g., NP uptake, NP intracellular localization, cell death mechanisms, oxidative stress, pro-inflammatory reactions, immunotoxicity or genotoxicity [10,15]. Nevertheless, criticisms have emerged regarding these mono-culture models, in that they are found to be too far from reality to allow proper evaluation of the complex interactions of NPs with the respiratory system [16]. Co-culture models have therefore been developed in order to better predict the toxicological effects of NPs in vivo. By associating various cell types such as epithelial, endothelial, macrophage, or dendritic cells, these co-culture models aim at closely mimicking cell–cell communications and cell–particle interactions occurring in the lung [9,17,18]. Unfortunately, some limitations have so far hampered the impact of co-culture models in NP safety assessment. First, such systems may be difficult to apply in screening or high-throughput assays due to more complex implementation and lower reproducibility as compared to standard monoculture models. Second, only few studies have currently compared the effects of NPs in a co-culture model *versus* the corresponding mono-culture models, in order to demonstrate the real added value of using such more sophisticated models [9,19,20,21]. Thirdly, due to technical constraints, most of the investigations carried out on co-cultures are descriptive cytotoxicity studies, without questioning the mechanisms involved in the NP-induced responses [9,10].

In this context, the first objective of the work reported herein was to establish and characterize a simple and robust co-culture model that mimics the deep lung. To do so, alveolar epithelial cells (A549 cell line) and macrophages (activated-THP-1 cell line) were selected. Although these cell lines are not normal which could be a limitation, they are widely used as in vitro airway models [21,22,23,24,25,26]. To set up an optimal model, A549 and THP-1 cells were seeded at several A549:THP-1 ratios, and the different established co-culture systems were characterized by determining their effective macrophage/epithelial cell ratio at the time of their use, and their capacity to respond to lipopolysaccharides (LPS), a well-known pro-inflammatory stimulus. After selecting the best co-culture model, our second objective was to determine the added value of this model in comparison with the respective mono-cultures for NP toxicity testing. To do so, we used carbon-based NPs called carbon dots (CDs), displaying easily tunable surface charge properties, and multiple biological endpoints, including NP cell uptake, oxidative stress, inflammation, and cell viability and death. CDs were discovered just over a decade ago [27] and are nanomaterials of tremendous interest to the scientific and industrial communities due to their unique and exciting properties, including ease of synthesis and functionalization, very small size (few nanometers), water solubility, intrinsic fluorescence and resistance to photobleaching [28]. Beside their applications in various fields of highly innovative and competitive industrial technologies, such as optoelectronics, photovoltaics and energy storage, chemical catalysis and chemical sensors, and anti-counterfeiting, CDs are currently being developed in nanomedicine for drug or gene delivery, biomedical imaging and theranostic applications [29,30,31,32]. Although they are generally described as biocompatible [33], a complete evaluation of their safety, however, is necessary mainly because of the large diversity of the synthesis methods that have been developed, leading to NPs with extremely variable physicochemical characteristics. Indeed, physicochemical characteristics of NPs, such as size, shape, chemical composition, or surface chemistry, are well known to impact their toxicological effects in the lung [34,35]. Among these characteristics, the surface charge is one of the most prominent factors of NP toxicity, with cationic NPs generally exhibiting greater toxicity than negative ones [36,37]. However, our group recently demonstrated that a cationic charge does not systematically confer toxicity to NPs [38]. In an original way, we have highlighted that the surface charge density of a cationic NP is more predictive of its toxicity than the absolute value of its zeta potential (ζ) [39]. Thus, in the present work, in order to reinforce these new data concerning the role of surface charge density in the toxicity of cationic NPs, we exposed our mono- and co-culture models to three cationic CDs exhibiting an increasing quantity of amino groups at their periphery, i.e., increasing surface charge density. These NPs were produced from citric acid in the presence of various amine group-containing passivation reagents, namely low-molecular-weight branched poly(ethylenimine) (MW = 600 Da, bPEI600), pentaethylene hexamine (PEHA), and N,N-dimethylethylene diamine (DMEDA). A negatively charged CD was also produced, through pyrolysis of ammonium citrate alone, and used as a control throughout our study. Our hypothesis was that the developed co-culture model of the human respiratory tract composed of epithelial cells and macrophages would allow to a more precise determination of the toxicological effects of NPs differing by their surface charge density than the respective mono-culture models.

## 2. Materials and Methods

### 2.1. Synthesis of CDs

The CDs investigated herein were produced according to the following procedures. Their surface charge density was tuned by synthetizing the NPs with passivation reagent containing an increasing number of amino groups (bPEI600 > DMEDA > PEHA).

CD-PEI600 and CD-PEHA. Citric acid (2.00 g), bPEI600 (8.00 g), and H_2_O (50 mL) were mixed in a beaker, and heated at 250 °C for 4 h, under continuous stirring. Small portions of water (5 mL) were periodically added to the mixture to prevent immobilization of the magnetic stirring bar. At the end of the process, the residue was dissolved in HCl 0.1 N, and loaded in a dialysis bag (MWCO 1000 Da) for dialysis against HCl 0.1 N (24 h) and ultra-pure water (24 h). The dialysate was filtered through a 0.22-μm polyethersulfone (PES) membrane and lyophilized to yield CD-PEI600 as an orange hygroscopic powder (1.63 g). CD-PEHA (1.41 g) was obtained similarly except that bPEI600 was replaced by PEHA (8.00 g).

*CD-DMEDA.* Citric acid (4.00 g), DMEDA (16.00 g), and water (10 mL) were mixed to homogeneity, introduced into a Teflon^®^-lined stainless-steel reactor, and heated at 210 °C for 72 h. The resulting solution was cooled to RT, transferred into a dialysis bag and treated as above to yield CD-DMEDA as a brown hygroscopic powder (1.11 g).

*CD-COOH.* Triammonium citrate (75.0 g) and KH_2_PO_4_ (15.0 g) in pure water (150 mL) were mixed in a beaker and heated at 250 °C for 1 h, under continuous stirring. The resulting dark residue was dissolved in HCl 0.1 N, filtered through a 0.45-μm PES membrane, and dialyzed. The dialysate was filtered through a 0.22-μm PES membrane and lyophilized to yield CD-COOH as a dark-brown powdered material (0.89 g).

### 2.2. Characterization of CDs

The hydrodynamic diameter of CDs was measured by dynamic light scattering (DLS, Zetasizer Nano ZS, Malvern Instruments, Paris, France) and calculated from the number distribution graph. Zeta potential was measured by DLS as well and calculated with the Smoluchowski’s equation. All measurements were performed in triplicate on fresh samples (1.0 mg/mL in 1.5 mM NaCl pH 7.4) at 25 °C. The surface charge density of CDs was determined by means of polyelectrolyte titration as described elsewhere [39]. In brief, the electrokinetic charge (Q_ek_) of the CDs was determined by monitoring zeta potential variation in the sample (1.0 mg/mL, NaCl 1.5 mM pH 7.4) alongside spiking with a solution of poly(acrylic acid) (PAA, MW ± 1800 Da, NaCl 1.5 mM pH 7.4). Q_ek_ was calculated from the amount of PAA required to bring ζ down to zero. The data were expressed in µmol of amine per mg of material. Optical properties of the CDs were characterized by carrying out UV-visible and fluorescence measurements on CD samples (1.0 mg/mL in 1.5 mM NaCl pH 7.4) using a Varioskan multimode reader (Thermo Scientific, Illkirch-Graffenstaden, France).

### 2.3. Cell Culture

The human alveolar epithelial cell line A549 (CCL-185^TM^, ATCC) was grown in DMEM/F12 culture medium containing 2 mM L-glutamine, 100 UI/mL penicillin, 100 µg/mL streptomycin, 5 mM Hepes and 10% fetal bovine serum (FBS). The human monocytes cell line THP-1 (TIB-202^TM^, ATCC) was grown in RPMI-1640 culture medium containing 2 mM L-glutamine, 0.05 mM 2-mercaptoethanol, 100 UI/mL penicillin, 100 μg/mL streptomycin, and 10% heat inactivated FBS (all reagents from GIBCO, Thermo Scientific, Illkirch-Graffenstaden, France). The two cell lines were grown in culture flasks at 37 °C in a 5% CO_2_ humidified chamber. For the experiments, the RPMI-1640 was used as culture medium in mono-cultures (A549 and THP-1) and co-cultures, after checking that medium change from DMEM-F12 (culture) to RPMI-1640 (experiment) did not impact the A549 cell growth. Co-cultures based on A549 and THP-1 cells have already been established in RPMI medium in the literature (e.g., Dekali et al. [21]).

### 2.4. Mono- and Co-Culture Models

**Figure 1:**
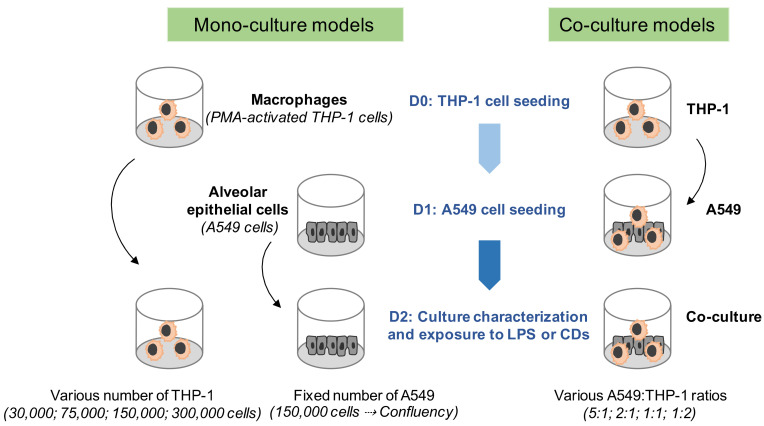
Schematic representation of the mono- and co-culture models used to assess the biological responses evoked by the CDs.

The culture models developed herein were adapted from previous studies combining A549 and THP-1 cells in co-culture [21,22,23,24,25,26]. Mono-cultures of activated THP-1 and A549 cells were prepared as follows. THP-1 cells were seeded into 24-well culture plates at a density of 30,000, 75,000, 150,000, or 300,000 cells/well in RPMI-1640 culture medium, and were differentiated into macrophages by incubation with 10 ng/mL phorbol 12-myristate 13-acetate (PMA, Sigma-Aldrich, St. Louis, MO, USA) for 24 h (Figure 1). Then, the cells were rinsed with phosphate-buffered saline (PBS) to remove PMA and further cultured in RPMI-1640 for 24 h before LPS or CD exposure. A549 epithelial cells were seeded into 24-well culture plates at a density of 150,000 cells/well in RPMI-1640 culture medium and exposed to LPS or CDs 24 h later (Figure 1). At this time, A549 cultures were at confluency. The co-cultures of THP-1 and A549 cells were prepared in parallel with monocultures (Figure 1). Briefly, THP-1 cells were seeded into 24-well culture plates at a density of 30,000, 75,000, 150,000, or 300,000 cells/well, and were differentiated into macrophages by incubation with 10 ng/mL PMA for 24 h. Then, the cells were rinsed with PBS to remove PMA, and A549 cells in RPMI-1640 medium were added at a density of 150,000 cells/well to obtain various A549:THP-1 ratios, namely 5:1, 2:1, 1:1, and 1:2. Co-cultures were grown for 24 h and characterized by flow cytometry or exposed to LPS or CDs as described below. Thus, the number of A549 cell in mono- and co-cultures was fixed and reached confluency whatever the culture model. Only the THP-1 number varied in order to modulate the proportion between the two cell types within the co-culture. The THP-1 number was modulated in the same way in the mono-culture models to allow comparison of the LPS- or CD-evoked biological responses between the mono- and co-culture models. Regarding the co-culture organization, the epithelial cells were located at the bottom of the wells and the macrophages often arranged into or on top of the epithelial cell layer.

### 2.5. Characterization of the Co-Culture Models by Fluorescence Activated Cell Sorting (FACS)

Co-cultures were rinsed with PBS, and cells were harvested by 0.05% trypsin treatment, transferred into microtubes and centrifuged (5 min, 200× *g*). The pelleted cells were rinsed with PBS containing 0.5% BSA before staining for 20 min with an APC-Cy7 anti-human CD14 antibody (M5E2 clone, 10 µg/mL, BioLegend, CA, USA) to identify THP-1-derived macrophages. After staining, cells were rinsed twice with PBS, cell suspensions were analyzed with a LSRFortessa X 20^TM^ flow cytometer (BD Biosciences, France), and collected data were analyzed using the FlowJo^TM^ software (v 10.2, Ashland, OR, USA). Macrophages were discriminated from epithelial cells according to their fluorescence intensity collected using an APC-Cy7 (red laser) channel. Macrophage number was expressed as a percentage of total cells inside the co-culture.

### 2.6. Cell Exposure to CDs

Once mono- and co-cultures were established, the media was removed and replaced with 400 µL of complete culture medium containing 0.01 µg/mL lipopolysaccharide (LPS, *Escherichia coli* O55:B5, Sigma-Aldrich, St. Louis, MO, USA) or CDs at the desired concentrations. Cells were exposed for 4 h before assessing NP internalization, cell death mechanisms and oxidative stress, and for 24 h before measuring cellular viability and pro-inflammatory cytokine production.

### 2.7. Cell Viability Assay

Changes in cell viability in response to CDs were assessed by the MTT (3-[4,5-dimethylthiazol-2-yl]-2,5 diphenyltetrazolium bromide) assay. Mono- and co-cultures were incubated with 400 µL of increasing concentrations of CDs (3–200 μg/mL, i.e., 0.6–40 µg/cm^2^) for 24 h. After this, cellular supernatant was removed and stored at −20 °C until cytokine assay, and cells were washed with PBS before addition of culture medium containing MTT (1.0 mg/mL, 300 μL). After incubation for 1 h, the MTT solution was removed, and cells were lysed with DMSO (500 µL). Absorbance of the resulting samples was read at 570 nm with a correction at 690 nm using a Multiskan FC reader (Thermo Scientific, Illkirch-Graffenstaden, France). Cell viability was expressed as the percentage of the absorbance of CD-treated cells relative to the absorbance of untreated cells.

### 2.8. Assessment of Cell Death Mechanisms

FACS was used to assess cell death mechanisms induced by CDs, as described by Grabowski et al. [24]. Mono- and co-cultures were incubated with 400 µL of 50 or 200 µg/mL (i.e., 10 or 40 µg/cm^2^) of CDs for 4 h. After CD exposure, the supernatant was discarded and cells were rinsed with PBS and harvested by 0.05% trypsin treatment. The cells were then stained with an APC-Cy7 anti-human CD14 antibody as previously described to distinguish macrophages from epithelial cells. Besides, to investigate the occurrence of apoptosis and/or necrosis, a doubled staining procedure using FITC-Annexin V (BioLegend, San Diego, CA, USA) and propidium iodide (BD Biosciences, Le Pont de Claix, France) was performed according to the manufacturer’s instructions. The fluorescence of each sample (30,000 events) was collected using an APC-Cy7 (red laser) channel (macrophage identification), as well as FITC (blue laser) and PE-Texas Red (yellow-green laser) channels (apoptosis and/or necrosis detection).

### 2.9. Oxidative Stress Assessment

Oxidative stress was assessed by FACS. Mono- and co-cultures were incubated with 400 µL of 50 or 200 µg/mL (i.e., 10 or 40 µg/cm^2^) of CDs for 4 h. After CD exposure, the supernatant was discarded and cells were rinsed with PBS and harvested by 0.05% trypsin treatment. The cells were stained with the APC-Cy7 anti-human CD14 antibody as previously described. Next, reactive oxygen species (ROS) production in response to CDs was measured using the fluorescent probe CellROX^TM^ Orange (Invitrogen^TM^, Thermo Scientific, Illkirch-Graffenstaden, France). Briefly, cells were incubated with culture medium containing 5 μM of the probe for 30 min and then rinsed twice with PBS before flow cytometry analysis. The fluorescence of each sample (30,000 events) was collected using an APC-Cy7 (red laser) channel (macrophage identification) and a PE (yellow-green laser) channel (ROS detection). Results were expressed as the fold change in fluorescence intensity of CD-exposed cells relative to the fluorescence intensity of non-exposed control cells.

### 2.10. Assessment of CD Cell Uptake

FACS and 3-D confocal laser scanning microscopy (CLSM) were used to assess CD uptake by cells, as described previously in the literature [24,26]. For FACS, mono- and co-cultures were incubated with 400 µL of 25 µg/mL (i.e., 5 µg/cm^2^) of CDs for 4 h. Then, the supernatant was discarded, cells were rinsed with PBS, harvested by 0.05% trypsin treatment, and stained with the APC-Cy7 anti-human CD14 antibody as previously described. The fluorescence of each sample (30,000 events) was collected using an APC-Cy7 (red laser) channel (macrophage identification) and a BV421 (violet laser) channel (CD detection). CD uptake was quantified by determining changes in the mean fluorescence intensity (MFI) of CD-exposed cells compared to the non-exposed control cells. For CLSM experiments, cells were seeded into 8-well IbiTreat μ-Slides (1.5 polymer coverslip, IBIDI^®^, Ibidi GmbH, Gräfelfing, Germany) and incubated with 25 µg/mL of CDs for 4 h. At the end of the incubation period, the cells were carefully washed with PBS containing 0.5% BSA before staining for 1 h with the Alexa Fluor^®^ 700-labeled anti-human CD14 antibody to identify THP-1-derived macrophages. After staining, cells were rinsed with PBS and the M488 fluorescent probe (200 nM in PBS) was added to the samples for 5 min to label the cell membrane [40]. Then, the intracellular localization of CDs was observed using a Leica SP2 microscope equipped with a 63× oil immersion objective (numerical aperture = 1.2). CDs, the anti-human CD14 antibody, and the M488 membrane probe were excited with 405, 635 and 488 nm laser sources, respectively.

### 2.11. Cytokine Assay

Interleukine-8 (IL-8) and monocyte chemoattractant protein 1 (MCP-1) were quantified in cell culture supernatants by ELISA according to the manufacturer’s instructions (R&D Systems, Lille, France).

### 2.12. Presentation and Statistical Analysis of the Data

Data were expressed as mean ± SEM and plotted as concentration-response curves or bar charts. Statistical differences between groups were determined by analysis of variance (ANOVA) followed by Tukey’s tests, using the GraphPad Prism software (v 6.0, San Diego, CA, USA). Data were considered as significantly different when the *p* value was less than 0.05.

## 3. Results and Discussion

### 3.1. Physicochemical Characterization of CDs

In this study, one CD exhibited a negative surface charge (CD-COOH, ζ = −43.3 ± 3.2 mV), and the other three were positively charged (CD-PEI600, ζ = +31.8 ± 1.1 mV; CD-PEHA, ζ = +29.2 ± 2.2 mV; CD-DMEDA, ζ = +11.1 ± 2.2 mV). The surface charge density of the cationic CDs increased with the nitrogen content of the passivation agent used for their synthesis, with the Q_ek_ value ranging from 0.01 µmol/mg for CD-DMEDA to 4.70 µmol/mg for CD-PEI600 (Table 1). The mean hydrodynamic diameter of the cationic CDs ranged from 10.2 to 28.7 nm, while the anionic CD-COOH had a mean hydrodynamic diameter of around 50 nm, revealing a stronger tendency to aggregate for this CD (Table 1). The CDs showed a UV-vis absorption peak at 350 nm, except for CD-DMEDA and CD-COOH, which absorbed decreasingly between 250 and 800 nm. The fluorescence emission and excitation spectra recorded on CD solutions revealed homogeneous optical properties between the four NPs, with a maximum fluorescence excitation and emission wavelengths at around 365 and 460 nm, respectively (Table 1). These intrinsic photoluminescence properties make it possible to analyze the cellular uptake of CDs with no conjugation to a fluorescent label required.

### 3.2. Characteristics of the Co-Culture According to the A549:THP-1 Seeding Ratio

**Figure 2:**
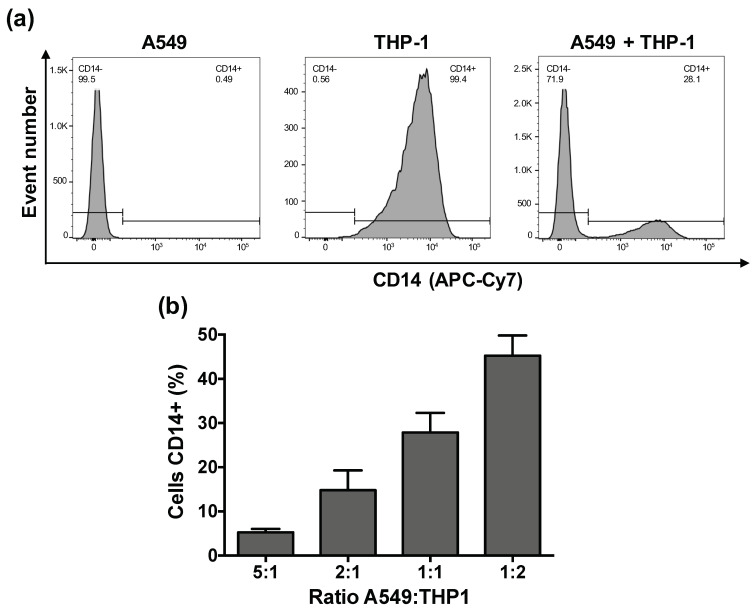
Determination of the proportion of macrophages in the co-culture models by FACS. (**a**) Staining and gating strategy to discriminate the A549 epithelial cells (CD14-) from the THP-1-derived macrophages (CD14+). (**b**) Proportion of macrophages (CD14+ cells) within the co-culture according to the A549:THP-1 seeding ratio two days after THP-1 seeding. Data are means ± SEM of n = 3 experiments.

Based on the literature, four A549:THP-1 seeding ratios were used to create our co-culture model: 5:1 [22], 2:1 [21,23,24], 1:1 [25], and 1:2 [26]. By modulating the proportion of epithelial cells to macrophages, it is possible to mimic physiological or inflammatory conditions. Indeed, in a healthy individual, 9.4% of lung cells are macrophages [41], whereas under pathological conditions or depending on individual lifestyle (smoker or not), the number of alveolar macrophages increases [42]. To characterize the four obtained models, we first quantified the proportion of macrophages inside the co-cultures at the time of their use, i.e., two days after THP-1 seeding (Figure 1), since the A549:THP-1 ratio may vary over time due to the proliferation of epithelial cells, unlike macrophages. To do so, we used a CD14 staining strategy that made it possible to discriminate the A549 epithelial cells (CD14 negative) from the THP-1-derived macrophages (CD14 positive) by FACS (Figure 2a). As shown on Figure 2b, the proportion of macrophages in our co-cultures increased when the A549:THP-1 ratio decreased, as expected, and reached 5%, 14%, 27%, and 45% for the initial A549:THP-1 seeding ratio of 5:1; 2:1; 1:1, and 1:2, respectively. Taking into account a proportion of 9.4% macrophages in the lung tissue of healthy individuals [41], in our study, the A549:THP-1 seeding ratio of 2:1 appeared to best mimic physiological conditions. This A549:THP-1 ratio of 2:1 is one of the most widely used in studies based on co-culture models [21,23,24]. However, although it is important to identify the real proportion of macrophages at the time of exposure to the respiratory toxicants, such a macrophage quantification within epithelial cell–macrophage co-culture models has been rarely reported in the literature [26].

**Figure 3:**
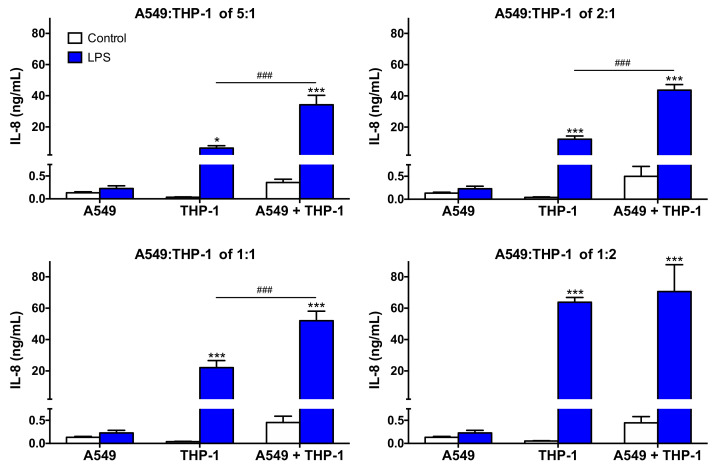
Capacity of the co-culture models to respond to a pro-inflammatory stimulus. Mono- and co-cultures were exposed to 0.01 µg/mL of LPS for 24 h, and the secretion of the pro-inflammatory cytokine IL-8 was measured in the culture supernatants. Data are means ± SEM of *n* = 3 experiments. Statistical differences at *p* < 0.05 (one symbol) and *p* < 0.001 (three symbols) were determined by ANOVA followed by the Tukey’s test when compared to control unexposed cells (asterisks) or to LPS exposed-THP-1 mono-culture (hash).

We then continued the characterization of our co-culture models by assessing their capacity to respond to LPS, a well-known pro-inflammatory stimulus. Thus, the secretion of the pro-inflammatory cytokine IL-8 was measured in the supernatants of mono- and co-cultures exposed to 0.01 μg/mL LPS (Figure 3). The response of the co-cultures was compared to that of the corresponding mono-cultures. After exposure to LPS, the level of IL-8 secretion was low in the A549 mono-culture (<500 pg/mL, not significant), whereas all the THP-1 mono-cultures secreted significant levels of the cytokine. As expected, in the THP-1 mono-cultures, IL-8 secretion induced by LPS increased as a function of cell number per well, reaching around 6500, 12,000, 22,000, and 63,000 pg/mL at the cell density of 30,000, 75,000, 150,000, and 300,000 cells/well, respectively. In the co-cultures, secretion of IL-8 in response to LPS was greater than in the corresponding mono-cultures, whatever the A549:THP-1 seeding ratio. Thus, the co-cultures were able to respond to a pro-inflammatory stimulus, which is consistent with the literature data [24,43,44,45]. Furthermore, the co-cultures more markedly responded to LPS compared to the corresponding THP-1 mono-cultures (*p* < 0.001), except at the A549:THP-1 seeding ratio of 1:2, suggesting a possible collaboration between epithelial cells and macrophages. Such a synergistic inflammatory response has already been described in the literature upon exposure of a A549:THP-1 co-culture to pathogens such as *Streptococcus pneumoniae* [44] or respiratory syncytial virus [46]. Some collaboration mechanisms between epithelial cells and macrophages have been proposed, which could involve activation of the NLRP3 inflammasome [21,47] or the TLR receptors [48]. Based on these characterization data, the co-culture model with the A549:THP-1 seeding ratio of 2:1 was selected to assess CD biological effects, as it better mimics the in vivo conditions in terms of the NP target cell ratio while appropriately responding to a pro-inflammatory stimulus.

### 3.3. Cytotoxicity of CDs and Cell Death Mechanisms in Mono- and Co-Cultures

**Figure 4:**
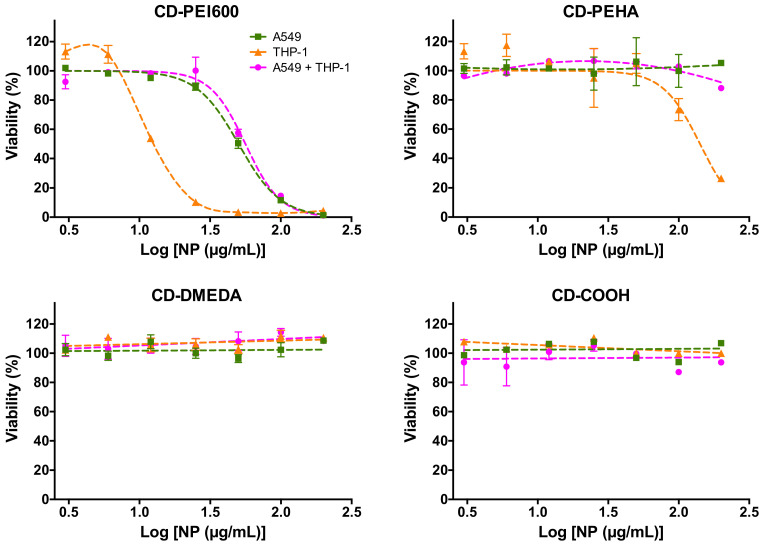
Cytotoxicity of the CDs in mono- and co-cultures. Mono- and co-cultures (A549:THP-1 seeding ratio of 2:1) were exposed to increasing concentrations (3–200 µg/mL) of CD-PEI600, CD-PEHA, CD-DMEDA, or CD-COOH for 24 h, and cell viability was assessed with the MTT assay. Results are expressed as the percentage of viability when compared to the control (unexposed cells). They are means ± SEM of *n* = 3–6 experiments. Concentration–response curves were obtained after logarithmic transformation of the data and fit with the Hill equation.

To evaluate CD cytotoxicity, mono- and co-cultures (A549:THP-1 at seeding ratio of 2:1) were exposed to increasing concentrations (3–200 µg/mL) of CD-PEI600, CD-PEHA, CD-DMEDA, or CD-COOH for 24 h, before assessing cell viability using the MTT assay. As shown in Figure 4, CD-PEI600 (ζ = +31.8 mV; Q_ek_ = 4.70 µmol/mg) induced a decrease in cell viability that reached 100% at the concentration of 200 µg/mL in both mono- and co-cultures. A loss of viability was also observed with CD-PEHA (ζ = +29.2 mV; Q_ek_ = 3.25 µmol/mg) but to a lesser extent and only in the THP-1 mono-culture. No cytotoxicity was observed for CD-DMEDA (ζ = +11.1 mV; Q_ek_ = 0.01 µmol/mg) and CD-COOH (ζ = −43.3 mV) in any cellular model. Thus, the anionic CD induced no cytotoxic effect and among the three cationic CDs, the one with the highest surface charge density exhibited the most potent toxicity whatever the culture model. In the literature, a greater toxicity of positive CDs compared to negative ones was reported [38,49,50]. However, our group also recently demonstrated that a positive zeta potential for NPs does not necessarily translate into toxicity, and that the surface charge density of NPs influences their toxicity as well [39], which is confirmed in this present study. Regarding the cellular models, the viability loss induced by CD-PEI600 and CD-PEHA was greater in THP-1 than in A549 mono-cultures. These data are in line with the literature reporting that macrophages are more sensitive to NP toxicity than lung epithelial cells [23,39,51,52]. This could be explained by the fact that macrophages internalize more NPs than epithelial cells, as we will discuss below. Surprisingly, when THP-1 cells were combined with A549 cells in a co-culture, no viability loss (CD-PEHA) or viability loss similar to that in the A549 mono-culture (CD-PEI600) was measured. These results are in keeping with the data reported by other groups on polystyrene [23] or silver [51] NPs, suggesting that the co-culture model would be less relevant than the macrophages in mono-culture for assessing the cytotoxicity of NPs. However, this hypothesis is based on data from the MTT colorimetric viability assay, which may be altered by disturbances in cell metabolism or interference with test compounds including NPs [53]. Besides, this method does not allow to discriminate of the viability of either cell type within the co-culture.

**Figure 5:**
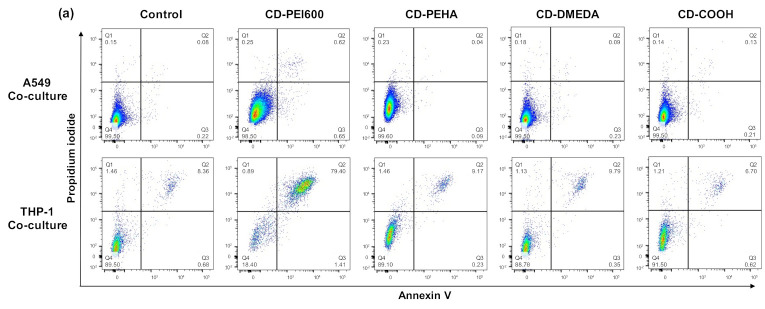

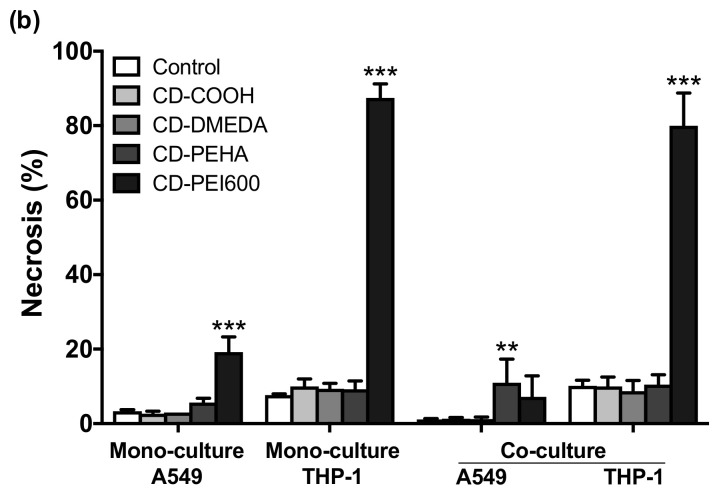
Cell death mechanisms evoked by CDs towards mono- and co-cultures. Mono- and co-cultures (A549:THP-1 seeding ratio of 2:1) were exposed to 50 µg/mL (CD-PEI600) or 200 µg/mL (CD-PEHA, CD-DMEDA and CD-COOH) for 4 h, and apoptosis/necrosis were assessed in each cell type by FACS. (**a**) Cell death mechanisms assessed by combined fluorescence analysis of the Annexin V apoptosis marker (X-axis) and propidium iodide necrosis marker (Y-axis) into A549 (CD14-) and THP-1 (CD14+) populations within the co-culture (data from *n* = 1 experiment). (**b**) Cellular mortality by necrosis in mono-culture and the co-culture, as expressed as the percent of double positive cells. Data are means ± SEM of *n* = 3–5 experiments. Statistical differences when compared to control unexposed cells were determined by ANOVA followed by the Tukey’s test. ** *p* < 0.01; *** *p* < 0.001.

To address this point, we assessed the cytotoxicity and the cell death mechanisms induced by the CDs in each cell type within the co-culture. This was performed through FACS experiments using the CD14 fluorescence labeling (Figure 2a). In the two identified cell populations, the cell death induced by CDs was assessed by combined fluorescence analysis of the annexin V apoptosis (X-axis) and propidium iodide necrosis markers (Y-axis) (Figure 5a). Mono-cultures were analyzed in the same way (data not shown). None of the CDs evoked cell apoptosis whatever the culture model. In contrast, as shown in Figure 5b, cationic CDs with the highest charge density (CD-PEI600) induced potent necrosis of THP-1 cells in the mono- and co-culture models, with no difference between the two models. Thus, macrophages were equally sensitive to the CD toxicity whether they were tested in mono- or co-culture. Besides, we showed that the macrophage cell death mechanism involved necrosis. In the literature, there are some conflicting data about the necrotic effects of NPs because most studies only report cell viability loss without focusing on cell death mode [54]. However, in line with our data, Wei et al. demonstrated that cationic NPs induce cell necrosis in alveolar macrophages from mouse lung and that this effect is dependent on the NP cationic surface charge [55]. In this study, cationic NPs (based on PEI or chitosan) lead to rapid necrosis through impairment of Na^+^/K^+^-ATPase. Another pathway involved in necrosis induced by NPs is NP degradation in lysosomes and subsequent destabilization of this organelle leading to the release of toxic substances into the cytosol and finally cell necrosis. Based on our previous studies on PEI-based CDs [56], we assume that the presence of many polyamine decorations at the surface of CD-PEI600 may promote such a toxicity mechanism in macrophages. In summary of this part, we showed that the cytotoxicity of CDs was dependent on their surface charge density. If the anionic CD did not induce any cytotoxic effect, we provided a ranking of the toxicity of the three cationic CDs that parallels their positive surface charge density, i.e., CD-PEI600 >> CD-PEHA > CD-DMEDA, with no toxicity of CD-DMEDA. In this cytotoxicity evaluation, the analysis of annexin V and propidium iodide markers by FACS was applicable to all cell models, in contrast to the MTT assay, which is better suited to mono-culture models. No added value of the co-culture model compared to macrophages in mono-culture was obtained.

### 3.4. Internalization of CDs in the Different Culture Models and Cell Types

**Figure 6:**
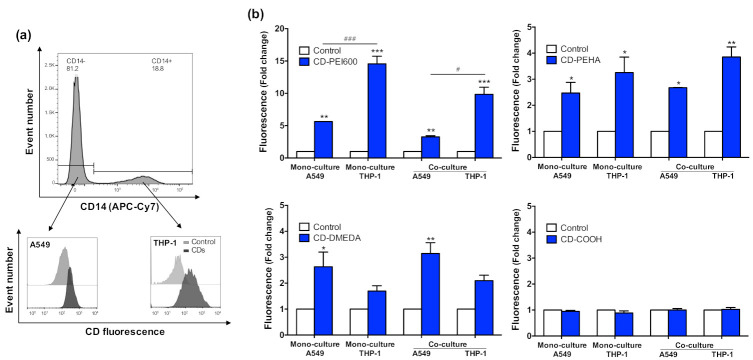

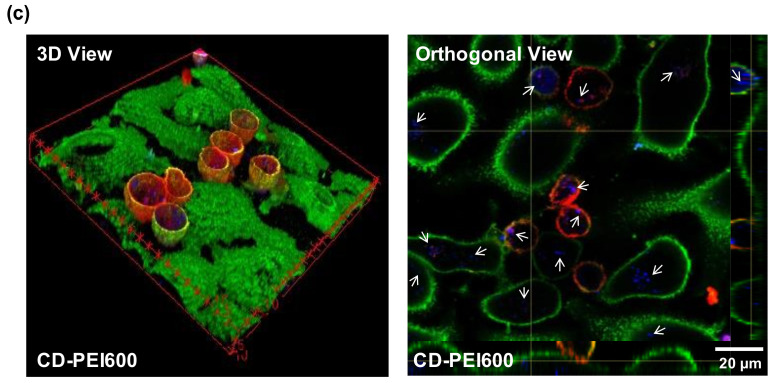
Uptake of CDs in the different culture models and cell types. Mono- and co-cultures (A549:THP-1 seeding ratio of 2:1) were exposed to 25 µg/mL of CDs for 4 h, and CD-uptake was assessed by FACS or 3-D CLSM. (**a**) Gating strategy to identify the two cell types present within the co-culture and to measure CD cell uptake by FACS after cell staining with an APC-Cy7 anti-CD14 antibody (data from *n* = 1 experiment). (**b**) Quantification of CD internalization by FACS. Results are expressed as fold change in fluorescence intensity when compared to control cells, and are means ± SEM of *n* = 3 experiments. Statistical differences at *p* < 0.05 (one symbol), *p* < 0.01 (two symbols) and *p* < 0.001 (three symbols) were determined by ANOVA followed by the Tukey’s test when compared to control unexposed cells (asterisks) or to CD-exposed A549 cells (hash). (**c**) 3D view (left, composite of full z-stack) and orthogonal view (right, xz and yz micrograph obtained from an optical slice of a z-stack) from CLSM observation of the co-culture exposed to 25 µg/mL of CD-PEI600 for 4 h. Cells were stained with the fluorescent membrane probe M488 and an anti-human CD14 antibody before observation. A549 cell membrane is colored in green, THP-1 cell membrane is colored in red, and CDs appear in blue and are indicated by white arrows (scale bar = 20 µm).

As cell uptake drives NP toxicity [57], we investigated the internalization of CDs by epithelial cells and macrophages in our different culture models. Mono- and co-cultures were exposed to 25 µg/mL of CDs for 4 h and CD uptake by A549 and THP-1 cells was assessed by monitoring CD-associated fluorescence by FACS and CLSM after cell staining with a cell membrane fluorescent probe (M488) and/or APC-Cy7 anti-CD14 antibody. As shown in Figure 6a,b, no fluorescence increase was observed in cells exposed to the anionic CD-COOH. In contrast, an increase in CD fluorescence signal was measured in A549 and THP-1 cells treated with cationic CDs in both mono- and co-culture models, with greater uptake of the highly positively charged CD-PEI600. Following observation of the co-culture by 3-D CLSM (Figure 6c), CD-PEI600 was visible as blue spots in the cytoplasm of epithelial cells (membrane in green) and macrophages (membrane in red), which confirmed the uptake of this cationic CDs by the two cell types within the co-culture model. Thus, a direct correlation was observed between the uptake of CDs and their toxicity profile, confirming the influence of the NP zeta potential [58] but also of the NP positive surface charge density [39] on these effects. In the literature, it has already been reported that a cationic surface charge could promote NP interactions with negatively charged serum proteins and therefore facilitate NP internalization by macrophages, in particular via phagocytosis [59]. However, whether the density of positive charges on the NP surface can impact the protein corona remains poorly explored [60] and will be an interesting focus for future research in the field.

Regarding the cellular models, we noted that CD-PEI600 uptake was higher in macrophages than in epithelial cells, both in mono- (*p* < 0.001) and co-cultures (*p* < 0.05). This result is in agreement with data from the literature on the internalization of PLGA [24], TiO_2_ and SiO_2_ [26], or silver [51] NPs in co-culture models associating lung epithelial cells and macrophages. Although investigating the uptake mechanisms of CDs was beyond the goal of the present work, we assume that the difference in CD uptake between the two cell types is presumably due to the phagocytosis mechanism involved in NP uptake by macrophages in contrast to epithelial cells [61]. Thus, in contrast to anionic CD-COOH, all three cationic CDs were internalized by the cells, in the following order: CD-PEI600 > CD-PEHA ≈ CD-DMEDA. These observations were similar whatever the cellular model used. By combining the FACS and CLSM approaches, our data thus showed that the co-culture model proposed in the present work is suitable for NP uptake studies and that macrophages are the main target of CDs in this model.

### 3.5. Oxidative Stress and Inflammatory Response Evoked by CDs in the Mono- and Co-Culture Models

**Figure 7:**
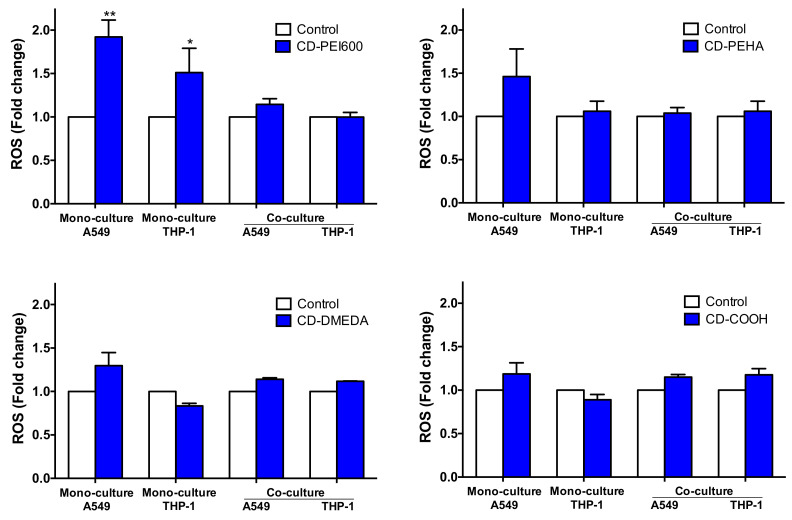
Oxidative stress evoked by CDs in mono- and co-cultures. Cultures were exposed to 50 µg/mL (CD-PEI600) or 200 µg/mL (CD-PEHA, CD-DMEDA and CD-COOH) for 4 h, and ROS were measured in A549 and THP-1 mono-cultures and in A549 (CD14- cells) and THP-1 (CD14+ cells) within the co-culture (A549:THP-1 seeding ratio of 2:1) by FACS using the fluorescent probe CellROX^TM^ Orange. Results were expressed as the fold change in the fluorescence intensity of CD-exposed cells relative to the intensity of the non-exposed control cells. Data are means ± SEM of *n* = 3 experiments. Statistical differences when compared to control unexposed cells were determined by ANOVA followed by the Tukey’s test. * *p* < 0.05; ** *p* < 0.01.

Oxidative stress is considered as a central mechanism of NP toxicity [62]. So, we investigated ROS production in mono- and co-cultures exposed to CDs for 4 h by FACS. As shown in Figure 7, the cationic CDs with the highest charge density (CD-PEI600), tested at the dose of 50 µg/mL, induced ROS production in A549 and THP-1 mono-cultures. None of the other CDs induced significant ROS production even at the highest tested dose of 200 µg/mL. Thus, cationic CDs with a high charge density have the capability of inducing ROS production in epithelial cells and macrophages, which could lead to cellular damages causing the necrosis that was observed. Surprisingly, no ROS increase in response to CD-PEI600 was measured in A549 and THP-1 cells when combined in the co-culture model. Although ROS production is a commonly used biomarker in nanotoxicology studies carried out on mono-culture models, it is still poorly monitored in co-culture models [20,43,63,64,65,66]. From the few reports found in the literature, it appears that NPs have no [66] or little impact on oxidative stress in co-culture models associating epithelial cells and macrophages [43,63,64]. This could be explained by the fact that the cellular interplay of the different cell types may help the cells dealing with oxidative stressors [20]. On the contrary, it has been recently reported that genes associated with antioxidant defense mechanisms (*NFKB1*, *KEAP1*, and *NFE2L2*) exerted slightly lower effects in the co-culture associating A549 and THP-1 cells as compared to the A549 mono-culture [65]. Assessing how antioxidant systems are modulated in response to CDs in mono- and co-culture models could be an interesting point to investigate in future work. Anyway, in this present study, we found no added value of the co-culture compared to mono-culture models in the oxidative stress assessment induced by CDs. Furthermore, ROS production for assessing the toxicological effects of CDs proved to be a low-sensitivity marker since among the four tested cationic CDs, only the CD with the highest charge density induced a change in ROS production.

**Figure 8:**
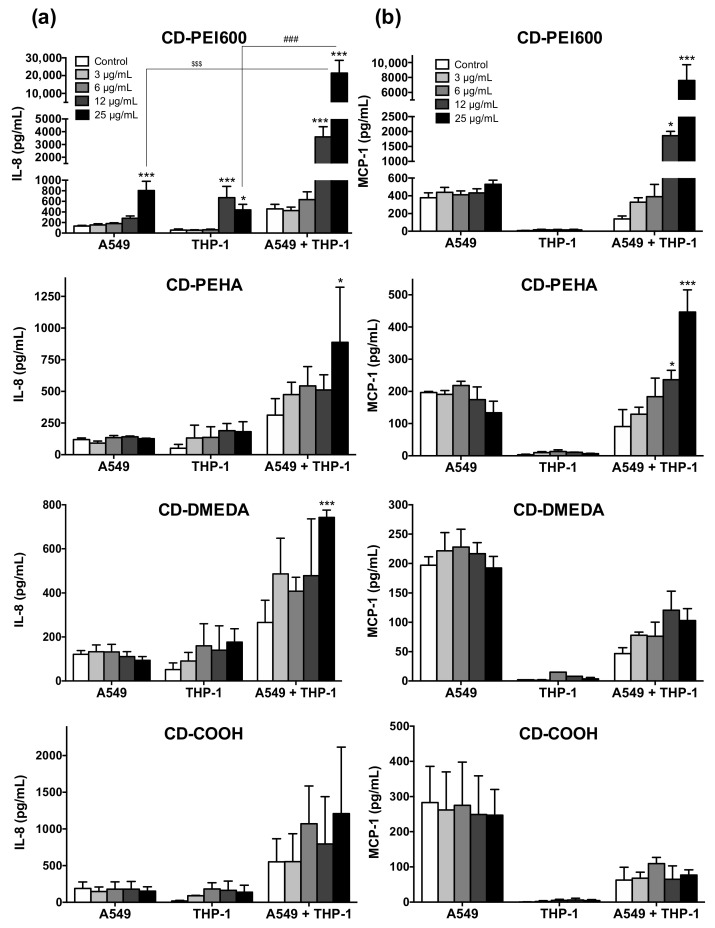
Inflammatory response evoked by CDs in mono- and co-cultures (A549:THP-1 seeding ratio of 2:1). Cultures were exposed to increasing concentrations (3–25 µg/mL) of CDs for 24 h and IL-8 (**a**) and MCP-1 (**b**) were quantified in cell culture supernatants. Data are means ± SEM of *n* = 3 experiments. Statistical differences at *p* < 0.05 (one symbol), *p* < 0.01 (two symbols) and *p* < 0.001 (three symbols) were determined by ANOVA followed by the Tukey’s test when compared to control unexposed cells (asterisks), to CD-exposed-THP-1 cells (hash), or to CD-exposed-A549 cells (dollar) in mono-cultures.

To investigate the inflammatory effect of CDs, the levels of IL-8 and MCP-1 were measured in the supernatants of mono- and co-cultures exposed to CDs at non cytotoxic concentrations (3–25 µg/mL, except for CD-PEI600 in the THP-1 mono-culture, Figure 4) for 24 h (Figure 8). The anionic CD-COOH did not induce any pro-inflammatory effect whatever the model. Regarding the cationic CDs, an IL-8 secretion was measured in the mono-cultures incubated with those with the higher charge density (CD-PEI600), but not with those of lower charge density (CD-PEHA and CD-DMEDA) (Figure 8a). In contrast, an IL-8 secretion was observed in response to all cationic CDs in the co-culture model, with the following ranking: CD-PEI600 > CD-PEHA > CD-DMEDA. In addition, only the co-culture model showed an increase in MCP-1 secretion after exposure to cationic CDs, with the same secretion ranking between CDs than with IL-8 (Figure 8b). Thus, contrary to what was observed for oxidative stress, an increased secretion of pro-inflammatory cytokines (e.g., 20-fold for IL-8 and 10-fold for MCP-1 with CD-PEI600) was evidenced in the co-culture model compared to mono-cultures, suggesting a collaboration mechanism between macrophages and epithelial cells in response to NPs. It has been suggested that such a collaboration mechanism could involve NLRP3 inflammasome [21,65] or activation of pro-inflammatory transcription factors [65]. In the literature, other studies using lung co-culture models reported significant pro-inflammatory effects of SiO_2_ [21,22], TiO_2_ [21,43], CeO_2_ [43], quartz [52], PLGA [24], polystyrene [67], and CuO [65] NPs. However, most of these studies either did not compare the inflammatory response measured in co-culture with mono-culture models or compared it only with epithelial cells and not with macrophages [43,52,65,67]. Among these studies, only Dekali et al. have actually demonstrated an increased inflammatory response in co-culture compared to epithelial cells and macrophages in mono-culture as we have [21]. In agreement with our data, the co-culture model of Dekali et al. was established with a A549:THP-1 seeding ratio of 2:1, suggesting that these culture conditions are well appropriate for evaluating NP-induced inflammatory responses [21]. Applied to our safety study on cationic CDs with increasing charge density, this is particularly relevant since only the co-culture model allowed us to find that CDs of medium (CD-PEHA) and low (CD-DMEDA) positive surface charge density could induce a pro-inflammatory response, with a greater effect for CD-PEHA compared to CD-DMEDA. If our previous work had already showed an inflammatory effect for cationic CDs with a high positive charge density [39], we report here for the first-time a pro-inflammatory potential for cationic CDs of lower charge density. By using the co-culture model developed in this work, we also showed that the inflammatory response induced by cationic CD is hierarchically related to the surface charge density of these NPs. Thus, we found a clear added value of the co-culture model compared to mono-cultures for NP-induced toxicological assessment.

## 4. Conclusions

In this study, we developed a simple co-culture model associating epithelial cells and macrophages that mimics the lung alveolar region. Using carbon-based NPs of different surface charge properties and multiple biological endpoints, we established some added value for this co-culture model in comparison to the respective mono-cultures for NP toxicity testing, as summarized in Table 2. Regarding the cytotoxicity, NP uptake, and oxidative stress endpoints, we did not find any advantage of the co-culture model over mono-cultures, especially macrophages. In contrast, our data highlighted a substantial contribution of the co-culture regarding inflammation assessment since this model allowed us to measure an inflammatory response for all tested cationic NPs. In addition, based on co-culture model data, we showed that the induced inflammatory response was hierarchically related to the surface charge density of cationic NPs. This underlines the major role of the surface charge properties in the toxicological responses of NPs, and the importance of considering the charge density, and not only the zeta potential, in the NP safety evaluation. To conclude, in our opinion, monitoring the inflammatory response in a co-culture model associating pulmonary epithelial cells and macrophages is the more powerful approach described thus far for discriminating as finely as possible the lung toxicological effects of NPs.

## Figures and Tables

**Table 1:** Physicochemical characteristics of the CDs investigated herein.

Characteristics	CD-PEI600	CD-PEHA	CD-DMEDA	CD-COOH
Zeta potential ζ (mV)	+31.8 ± 1.1	+29.2 ± 2.2	+11.1 ± 2.2	−43.3 ± 3.2
Surface charge density Q_ek_ (µmol/mg)	4.70	3.25	0.01	-
Hydrodynamic diameter D (nm)	11.0 ± 3.4	10.2 ± 3.2	28.7 ± 4.1	50.7 ± 0.9
Photoluminescence λ_max_/λ_ex_/λ_em_ (nm)	350/365/460	350/370/465	^a^/315/465	^a^/370/445

^a^: Monotone and decreasing UV-vis absorption between 250 and 800 nm.

**Table 2:** Overview of the data.

		Viability loss	Necrosis	Uptake	ROS	IL-8	MCP-1
**A549**	CD-PEI600	+	+	+	+	+	-
	CD-PEHA	-	-	+	-	-	-
	CD-DMEDA	-	-	+	-	-	-
	CD-COOH	-	-	-	-	-	-
**THP-1**	CD-PEI600	+	+	+	+	+	-
	CD-PEHA	+	-	+	-	-	-
	CD-DMEDA	-	-	-	-	-	-
	CD-COOH	-	-	-	-	-	-
**A549+THP-1**	CD-PEI600	+	+	+	-	+	+
	CD-PEHA	-	-	+	-	+	+
	CD-DMEDA	-	-	+	-	+	-
	CD-COOH	-	-	-	-	-	-

+: significant effect; -: no significant effect.

## Data Availability

Data presented in this study are available by requesting from the corresponding author.
